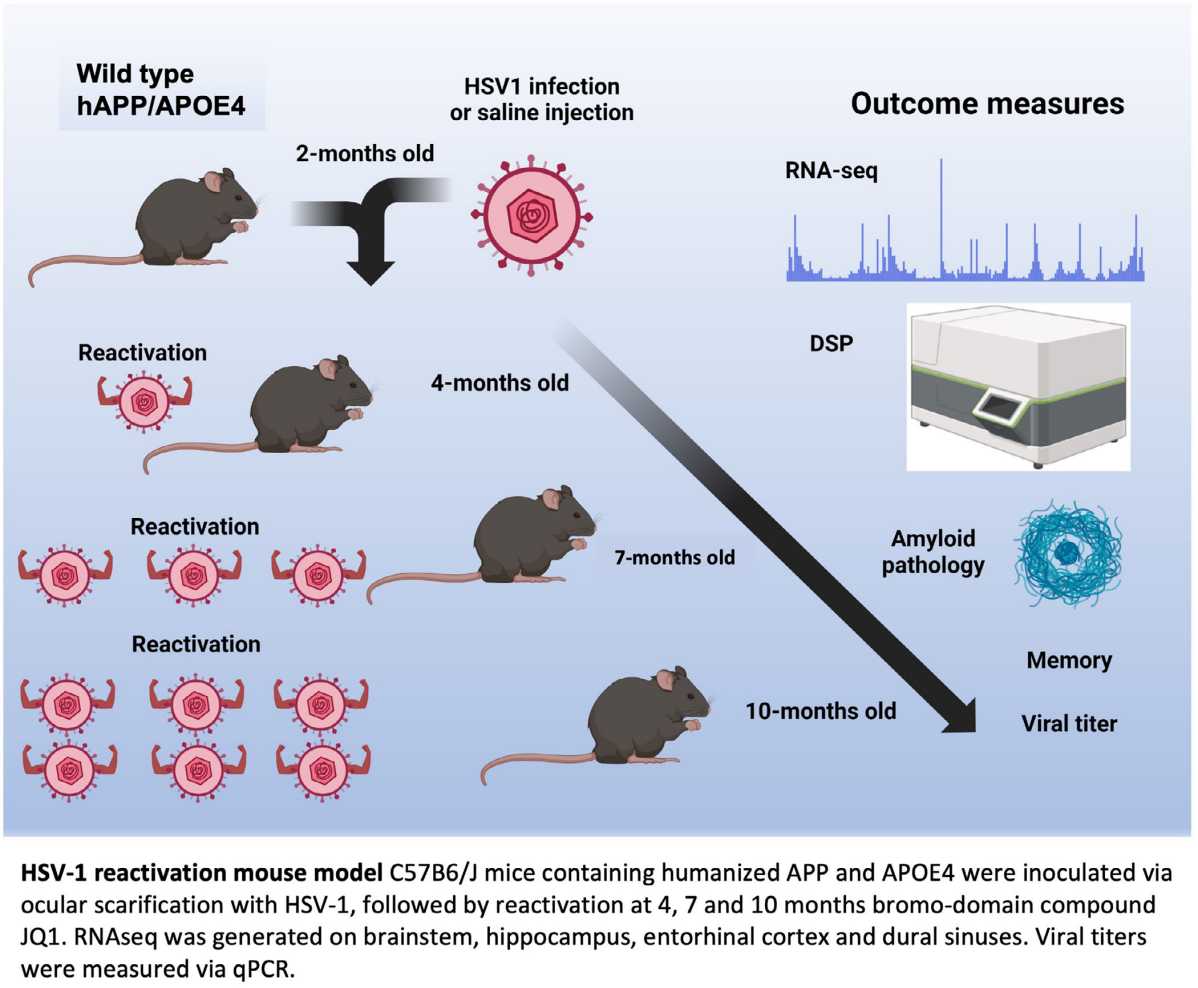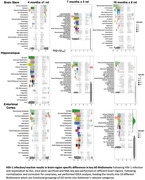# Repeated reactivation of HSV‐1 in mouse brain spreads radially from the trigeminal ganglion, altering expression of synaptic and immune genes in patterns similar to early AD

**DOI:** 10.1002/alz.093497

**Published:** 2025-01-03

**Authors:** Cory C Funk, Martin Darvas, Christine Johnston, Max Robinson, Caitlyn Schaffer, Christian Battaglia, Martine Aubert, Jesse C Wiley

**Affiliations:** ^1^ Institute for Systems Biology, Seattle, WA USA; ^2^ University of Washington School of Medicine, Seattle, WA USA; ^3^ University of Washington, Seattle, WA USA; ^4^ Fred Hutch Cancer Center, Seattle, WA USA; ^5^ Sage Bionetworks, Seattle, WA USA

## Abstract

**Background:**

The immerging role of CD8+T cells, interferon and the adaptive immune response in AD is consistent with previous observations of the putative role of neurotrophic herpesvirus family infections contributing to Alzheimer’s Disease pathophysiology. An outstanding question is how chronic viral infections over decades may contribute to AD pathogenesis. Our HSV‐1 reactivation model aims to provide insights to this question.

**Method:**

We infected 2 month old C57B6/J mice containing humanized APP and APOE4 with 10^6^ PFU HSV‐1 strain 17 via corneal scarification. We reactivated the virus with the bromo‐domain compound JQ1 at 4, 7 and 10 months, with 1x, 3x and 6x reactivations respectively and harvested the brainstem, hippocampus, entorhinal cortex and dural sinuses, for RNAseq. To identify common changes in gene expression between our HSV‐1 reactivation model and AD, we compared the gene expression profiles of our mice to both human and mouse model gene profiles using 19 AD‐specific categories (BioDomains).

**Result:**

We found reactivation by JQ1 resulted in increased HSV‐1 detection by qPCR in a progressive manner, emanating outward from the trigeminal ganglion, towards more distal brain regions. We observed changes in RNAseq expression profiles in a time‐dependent manner, with the greatest number of differentially expressed genes at 7 months. We observed changes in synaptic and immune genes in both the entorhinal cortex and hippocampus. We also saw decreases in gene expression in lipid metabolism, myelination, and the endolysosomal pathways.

**Conclusion:**

Our HSV‐1 reactivation mouse model identifies changes in gene expression that are similar to those observed in humans and mouse AD models, with observed, distinct changes in synaptic‐ and immune‐associated genes in multiple brain regions. Activation of immune genes was highest in the brain stem, where detection of HSV‐1 was highest. The greatest changes in gene expression occurred at 7 months, with fewer changes following at 10 months. These changes are most similar to changes in early disease and support the role of HSV‐1 contributing to AD pathophysiology.